# Renal functional outcomes after minimally invasive partial nephrectomy, single‐surgeon experience

**DOI:** 10.1002/bco2.70268

**Published:** 2026-08-18

**Authors:** Mohamed Elsaqa, Ahmed Rabie, Israel Franco, Andrew Fishman, Wayne Debeatham, Michael Grasso

**Affiliations:** ^1^ Department of Urology Phelps Hospital, Northwell Health Sleepy Hollow New York USA; ^2^ Faculty of Medicine Alexandria University Alexandria Egypt; ^3^ Yale School of Medicine New Heaven Connecticut USA; ^4^ Department of Urology New York Medical College Valhalla New York USA

**Keywords:** eGFR, partial nephrectomy, RCC, renal mass, warm ischaemia time

## Abstract

**Objectives:**

This study aimed to report the impact of minimally invasive partial nephrectomy (PN) on short‐ and long‐term renal functional outcomes through a single‐institution, long‐term experience.

**Materials and methods:**

We retrospectively reviewed consecutive patients who underwent retroperitoneal laparoscopic and/or robotic‐assisted PN for renal masses by a single surgeon between 2016 and 2025. A multimodal perioperative renal‐protective protocol was routinely utilized. Baseline estimated glomerular filtration rate (eGFR) was compared with early and late postoperative eGFR changes surveilled over a 2‐year period post‐PN. Multivariate regression analysis of multiple variables was performed for the association with change in eGFR at 2‐year follow‐up.

**Results:**

A total of 281 patients with a mean (standard deviation [SD]) age of 67.70 (±10.05) years were included. Mean (SD) baseline serum creatinine and eGFR were 1.04 (±0.31) mg/dL and 74.08 (±19.28) mL/min, respectively. Mean eGFR showed an early postoperative significant decline to 63.98 ± 19.8 mL/min at 2 weeks, followed by delayed recovery at 6 months (69.23 ± 20.87) and then stabilization through 1‐ (69.4 ± 19.26) and 2‐year (68.15 ± 19.67) follow‐up. Multivariate logistic regression analysis identified renal mass size (*p* < 0.001, odds ratio [OR] = 4.78, 95% confidence interval [CI]: 3.34 to 6.23) and preop eGFR (*p* < 0.001, OR = 0.13, 95% CI: 0.06 to 0.2) as the independent significant predictors of functional decline at 2‐year follow‐up, whereas other variables including warm ischaemia time (WIT) were statistically insignificant.

**Conclusion:**

After PN, renal function demonstrates a pattern of early decline followed by substantial recovery over time. Tumour size and preoperative eGFR were the main predictors of late eGFR changes. With proper surgical techniques and perioperative renal‐protective strategies, the effect of WIT can be mitigated.

## 1 INTRODUCTION

Nephron‐sparing surgery via partial nephrectomy (PN) represents the standard of care for localized renal masses, providing oncological outcomes comparable to radical nephrectomy while preserving renal function.^1^, ^2^ The widespread adoption of minimally invasive PN using laparoscopic and robotic‐assisted approaches has improved perioperative outcomes, including reduced blood loss and shorter hospital stay.^3^, ^4^

Warm ischaemia time (WIT) during PN has been considered a key determinant of postoperative renal functional outcomes.^5^, ^6^ Studies have demonstrated that increasing WIT is associated with a stepwise decline in postoperative estimated glomerular filtration rate (eGFR).^7^, ^8^ Historically, a threshold of 20–30 min has been considered acceptable.^9^, ^10^ Surgical technical innovations such as early unclamping, selective clamping and zero‐ischaemia techniques have been developed to minimize ischaemic insult.^11^, ^12^, ^13^ On the contrary, some recent studies have challenged this paradigm and reported less effect of WIT on renal function post‐PN compared to other factors such as preserved renal parenchymal volume.^14^, ^15^ Studies have proposed that factors affecting renal function post‐PN include older age, female gender, high body mass index (BMI), low preoperative eGFR, solitary kidney, large tumour size and increased mass complexity.^16^, ^17^

In the present study, we aimed to report the impact of WIT on short‐ and long‐term renal function and assess the predictors of renal function loss after minimally invasive PN.

## 2 METHODS

This retrospective cohort study included consecutive patients who underwent laparoscopic and/or robotic‐assisted laparoscopic PN for renal masses by a single surgeon (M. G.) between September 2016 and January 2025. The study included adult patients (≥18 years) who underwent minimally invasive PN with hilar clamping under warm ischaemia with complete follow‐up of at least 1 year. Patients with incomplete clinical data or missing follow‐up renal function measurements were excluded from the analysis. Institutional Review Board (IRB) approval was obtained prior to data collection.

### 2.1 Surgical technique

All procedures were performed using laparoscopy and/or robotic‐assisted laparoscopy through a retroperitoneal approach using a 4‐port technique. The shift from pure laparoscopy to robotic‐assisted PN was made in 2020. After identification and meticulous dissection of the renal hilum, tumours were always identified and boundaries marked utilizing intraoperative ultrasound. Vascular control of the renal artery was achieved using two non‐crushing bulldog clamps without clamping of the renal vein. Tumour excision was performed with the enucleation technique in most of the cases. Renorrhaphy was performed with efforts made to minimize WIT in all cases.

### 2.2 Perioperative renal‐protective protocol

A standardized perioperative protocol aimed at optimizing renal perfusion and minimizing ischaemia‐related injury was implemented in all PN patients. This protocol included the following:

- *Preoperatively*, angiotensin‐converting enzyme inhibitors (ACEIs) or angiotensin‐receptor blockers (ARBs) were discontinued at least 1 week prior to surgery.
- *Intraoperatively*, before renal artery clamping, patients were well hydrated with intravenous fluids and then administered intravenous 20 mg of furosemide or mannitol to enhance renal blood flow. Additionally, with the aim of preventing renal vascular thrombi, low‐dose intravenous heparin (2000 units) was routinely administered approximately 5 min before arterial clamping. If bleeding was encountered, it was quickly reversed using protamine sulphate administered intravenously.
- *Postoperatively*, nephrotoxic medications were avoided.

### 2.3 Data collection

Preoperative patient demographics, baseline renal function, tumour characteristics, intraoperative details and early and late postoperative outcomes and complications were collected and analysed. eGFR was calculated using the Chronic Kidney Disease Epidemiology Collaboration (CKD‐EPI) creatinine equation. The primary clinical outcome under investigation was the percentage change between preoperative baseline eGFR and eGFR at 2‐year follow‐up, defined as ‘Delta eGFR’ or ‘∆ eGFR’. Multivariate regression analysis of multiple variables was performed to assess the association with ∆ eGFR.

### 2.4 Statistical analysis

Categorical data were summarized as numbers and percentages, whereas quantitative data were described as mean ± standard deviation (SD) or median with interquartile range (IQR). The Shapiro–Wilk test was used to test for normality of continuous variables. The significance of the results was judged at the 5% level. Paired *T*‐test and analysis of variance (ANOVA) were used to compare quantitative variables (eGFR) at different time points. A multivariate regression analysis model was used to predict factors associated with ∆ eGFR. A linear regression model was used to evaluate the effect of mass size, blood loss and ischaemia time. A linear mixed‐effects model was used to analyse the eGFR data. Data were analysed using IBM SPSS software package version 27.0 (Armonk, NY: IBM Corp, released 2020).

## 3 RESULTS

A total of 281 patients with complete charts and follow‐up data were included in the study. The mean (SD) age was 67.70 (±10.05) years, with an American Society of Anesthesiologists (ASA) score of III in 176 (62.7%) patients. Mean (SD) baseline creatinine was 1.04 (±0.31) mg/dL, and mean (SD) eGFR was 74.08 (±19.28) mL/min. Median (IQR) preoperative renal mass size was 3 (2.2–3.4) cm. The cohort included five (1.8%) patients with solitary kidneys. Patients' demographics, comorbidities, baseline renal function and renal mass characteristics are detailed in Table 1.

**TABLE 1:** Baseline patient characteristics and perioperative data.

Parameter		Overall cohort (*n* = 281)
Age, years, mean (SD)		67.70 (10.05)
Sex, *n* (%)	Male	166 (59%)
	Female	115 (41%)
Weight, kg, mean (SD)		85.76 (17.91)
BMI, kg/m^2^, mean (SD)		29.53 (6.71)
Diabetes mellitus, *n* (%)		66 (23.4%)
Hypertension, *n* (%)		184 (65.4%)
Cardiovascular disease, *n* (%)		83 (29.54%)
ASA score, *n* (%)	II	105 (37.4%)
	III	176 (62.7%)
Baseline creatinine, mg/dL, mean (SD)		1.04 (0.31)
Baseline eGFR, mL/min/1.73 m^2^, mean (SD)		74.08 (19.28)
Renal mass side, *n* (%)	Right	145 (51.6%)
	Left	136 (48.4%)
Renal mass size, cm, median (IQR)		3 (2.2–3.4)
Renal mass location, *n* (%)	Upper pole	98 (34.9%)
	Mid‐pole	92 (32.7%)
	Lower pole	82 (29.2%)
	Hilar	9 (3.2%)

Abbreviations: ASA, American Society of Anesthesiologists; BMI, body mass index; eGFR, estimated glomerular filtration rate; IQR, interquartile range; SD, standard deviation.

Laparoscopic PN was performed in 238 patients, whereas 43 patients had robotic‐assisted PN. None of the cases required conversion to open surgery. Mean ± SD intraoperative WIT was 19.47 ± 8.6 min, with median estimated blood loss of 100 mL. Only seven (2.49%) patients required blood transfusion; four of them had low preoperative haemoglobin levels. Clavien–Dindo grade ≥III complications occurred in only five (1.8%) of patients. Minor (grade I and II) complications included small pneumothorax, transient acute kidney injury (AKI), ileus, leucocytosis and postoperative heart rate changes that were managed conservatively, whereas grade III complications included chest tube insertion for pneumothorax in three patients and percutaneous drainage of urinoma collections in two patients. Pathology was benign (oncocytoma and angiomyolipoma [AML]) in 45 (16%) patients. In 23 (8%) patients, an upgrade from clinical T1 to pathological T3a renal cell carcinoma (RCC) was seen due to microscopic perinephric fat infiltration or segmental vein invasion in 12 and 10 patients, respectively (Table 2). With a median follow‐up of 46 months, none of the patients developed tumour bed recurrence, whereas 24 (8.4%) patients had metachronous ipsilateral or contralateral renal masses.

**TABLE 2:** Perioperative outcomes of the study population.

Parameter		Result
Ischaemia time, min, mean ± SD		19.47 ± 8.6 min
Estimated blood loss, mL, median (IQR)		100 (50–150)
Haemoglobin drop, g/dL, mean ± SD		0.88 ± 0.55
Hospital stay, days, median (IQR)		2 (2–3)
Blood transfusion, *n* (%)		7 (2.49%)
Pathological tumour size, cm, median (IQR)		2.8 (2.5–3.5)
Pathological T stage	T1a	243 (86.48%)
	T1b	15 (5.34%)
	T3a	23 (8.2%)
Histopathological type, *n* (%)	Clear cell RCC	156 (55.5%)
	Chromophobe RCC	40 (14.23%)
	Papillary RCC	34 (12.1%)
	Oncocytoma	38 (13.5%)
	AML	7 (2.5%)
	Other	6 (2.1%)
Clavien–Dindo classification, *n* (%)	Grade I	17 (6%)
	Grade II	15 (5.3%)
	Grade III	5 (1.8%)

Abbreviations: AML, angiomyolipoma; IQR, interquartile range; RCC, renal cell carcinoma; SD, standard deviation.

Mean eGFR ± SD showed an early postoperative significant decline from baseline of 74.08 ± 19.28 to 63.98 ± 19.80 mL/min at 2 weeks postoperatively (*p* < 0.001). Functional stagnation persisted through the 3‐month milestone (mean eGFR 64.4 ± 17.82 mL/min). However, delayed recovery was observed between the 3‐ and 6‐month windows, with the adjusted mean eGFR at 6 months recovering to 69.23 ± 20.87 mL/min (*p* < 0.001). Beyond the 6‐month mark, no statistical differences were found between the 6‐month, 1‐year (mean: 69.4 ± 19.26) and 2‐year (mean: 68.15 ± 19.67) milestones (*p* > 0.05) (Table 3).

**TABLE 3:** Changes in the estimated GFR.

	Pre‐operative (*n* = 281)	After 2 weeks (*n* = 281)	6‐month follow‐up (*n* = 267)	2‐year follow‐up (*n* = 183)
eGFR, mL/min, mean (SD)	74.08 (19.28)	63.98 (19.80)	69.23 (20.87)	68.15 (19.67)
eGFR change, mL/min, mean (SD)		9.6 (−5.0 to 17)	3 (−4 to 10)	5.93 (−7 to 20)
% of change, median (IQR)		10.4 (−1.7 to 21.4)	3.67 (−7.41 to 14.29)	7.69 (−2.13 to 16.6)

Abbreviations: eGFR, estimated glomerular filtration rate; IQR, interquartile range; SD, standard deviation.

To identify independent predictors of ultimate renal function, a multivariable linear regression model was constructed using Delta eGFR (∆ eGFR) as the continuous dependent outcome variable. When adjusting simultaneously for patient baseline demographics and intraoperative metrics, mass size (*p* < 0.001) and preop eGFR (*p* < 0.001) emerged as the dominant, highly significant independent continuous predictors of functional decline. In other words, for every 1‐cm increase in resected tumour diameter, ∆ eGFR worsened by 4.78 units (*B* = 4.78, 95% confidence interval [CI]: 3.34 to 6.23, *p* < 0.001). Similarly, a higher baseline functional reserve was independently associated with a lower absolute postoperative ∆ eGFR, with every 1‐unit increase in preop eGFR corresponding to a 0.13‐unit drop in absolute ∆ eGFR (*B* = 0.13, 95% CI: 0.06 to 0.20, *p* < 0.001). Other factors including WIT (*p* = 0.065), estimated blood loss (*p* = 0.19), BMI (*p* = 0.376), hypertension (HTN) (*p* = 0.325) and diabetes mellitus (DM) (*p* = 0.488) did not significantly influence ∆ eGFR.

A sub‐analysis was performed to compare renal functional outcomes in robotic‐assisted versus pure laparoscopic cases. The baseline demographics and renal function were comparable. Mean WIT in laparoscopic versus robotic‐assisted groups was 19.41 and 16.09 min, respectively; this difference trended towards but did not reach statistical significance (*p* = 0.072). Pairwise comparisons demonstrated no statistically significant differences in eGFR between the laparoscopic and robotic groups at baseline (*p* = 0.614), 2‐week (*p* = 0.777), 3‐month (*p* = 0.923), 6‐month (*p* = 0.498) or 1‐year follow‐up (*p* = 0.622).

## 4 DISCUSSION

PN has been the standard management option for localized renal masses with superior renal functional preservation outcomes over radical nephrectomy.^18^ The present study evaluated perioperative and renal functional outcomes of minimally invasive PN in a contemporary cohort of 281 patients. The study demonstrated a characteristic biphasic renal function trajectory following PN: an acute significant decline at 2 weeks postoperatively followed by partial recovery to near‐baseline levels by 3–6 months, with functional stability thereafter through 2 years of follow‐up. This confirms that the early nadir reflects a reversible functional insult rather than permanent nephron loss. The fact that eGFR values at 6 months through 2 years did not differ significantly from the preoperative baseline is clinically reassuring as it indicates that the impact of WIT is transient and consistent with the nephron‐sparing principle that PN can preserve the majority of functional renal mass over time. The results are consistent with prior studies that reported significant early decline in eGFR followed by gradual improvement over time.^19^, ^20^

The current study has shown a consistent association between tumour size and postoperative renal functional outcomes. In multivariable linear regression, mass size was an independent predictor of absolute GFR decline (*B* = 4.78, 95% CI: 3.34 to 6.23, *p* < 0.001). The choice of a proportional rather than absolute GFR decline threshold was methodologically important, as absolute metrics systematically underestimate the functional burden experienced by patients with pre‐existing renal impairment. These findings align with the paradigm shift that has occurred over the past decade in understanding the drivers of post‐PN renal function.

Warm ischaemia has long been considered a critical determinant of renal functional outcomes after PN. Foundational studies have demonstrated a direct relationship between ischaemia duration and renal injury.^5^, ^6^ However, more contemporary evidence has challenged this paradigm, suggesting that the impact of warm ischaemia may be overestimated when considered in isolation. Large observational studies and systematic reviews have demonstrated that within commonly observed clinical ranges, the duration of warm ischaemia may not be the dominant predictor of long‐term renal function.^8^, ^21^, ^22^ Our data support this evolving perspective, as multivariate analysis showed no independent association between WIT and renal function decline.

Tumour size emerged as an independent predictor of significant eGFR reduction. This observation is consistent with prior studies emphasizing the importance of tumour complexity and parenchymal preservation over ischaemia time alone. Ginzburg et al. demonstrated that the quantity of preserved renal parenchyma is the strongest determinant of renal function at 6 months post‐PN, outweighing the effect of WIT.^15^ Similarly, Lane et al. and Simmons et al. reported that functional outcomes after PN are more closely related to nephron mass preservation and baseline renal function than to ischaemia time itself.^23^, ^24^ From a mechanistic standpoint, larger tumours are associated with increased surgical complexity, greater parenchymal loss and more extensive reconstruction, all of which may contribute to renal functional decline. These factors may explain why tumour size, rather than ischaemia duration, emerged as the primary predictor in our cohort. Importantly, this finding reinforces the concept that surgical quality and parenchymal preservation remain the cornerstone of nephron‐sparing surgery. From the same perspective, studies have reported more preserved renal parenchymal volume and better renal functional outcomes with the tumour enucleation technique, which dissects the mass along the pseudo‐capsule plane, compared to the standard PN technique, which excises the mass plus a rim of normal parenchyma.^25^, ^26^

A 2025 systematic review and meta‐analysis by Li et al., encompassing about 5000 patients across 10 studies, found that extended warm ischaemia was associated with decreased postoperative eGFR (odds ratio [OR] = 1.08; 95% CI: 1.02 to 1.15; *p* = 0.006) but critically noted that the threshold of potentially harmful ischaemia time ranged between 25 and 30 min, with limited ischaemia below this threshold demonstrating minimal additional risk compared to zero‐ischaemia techniques.^7^ In the present cohort, the mean WIT of 19.4 min among patients undergoing ischaemic techniques falls below this reported injury threshold, which may explain why ischaemia time did not emerge as an independent driver of GFR decline after accounting for other covariates. Similarly, Rosen et al. demonstrated in a multi‐institutional robotic PN cohort that even variations in shorter WIT below 20 min could affect AKI rates, but the long‐term functional signal was substantially attenuated once excisional volume loss was controlled.^27^

The percentage change in eGFR at 2 years post‐PN in our results, ‘∆ GFR’, also correlated with the preoperative eGFR, a finding consistent with a previous report by Simmons et al., who reported that the early drop in eGFR correlated with WIT and the late drop in eGFR correlated with the preserved renal volume, whereas both the early and late drops in eGFR correlated with preoperative eGFR.^24^ This can be explained by lower functional reserve in patients with pre‐existing renal impairment, whereby even a modest absolute loss in GFR may constitute a proportionally larger and more clinically consequential reduction.

In the current study, robotic‐assisted PN and laparoscopic PN were comparable regarding WIT and eGFR change outcomes. On the contrary, Pierorazio et al. reported favourable outcomes with robotic‐assisted PN through shorter operative time, shorter WIT and lower estimated blood loss compared to laparoscopic PN.^28^

The current study provides consistent important clinical insights through a large cohort of minimally invasive PN with long‐term follow‐up of 2 years. To the best of our knowledge, our study is the first to report complete 2‐year follow‐up renal function post‐PN. The study has some limitations that include being a retrospective single‐institution study that may limit generalizability to other surgical settings, patient populations or centres with different case volumes or technical approaches. Also, the dataset does not include volumetric measures of parenchymal preservation such as functional parenchymal volume or the percentage of parenchyma resected. Tumour diameter was used as a surrogate for resection volume, which, while validated in multiple prior studies, does not account for tumour endophytic depth, proximity to the collecting system or reconstructive parenchymal loss. Furthermore, the favourable functional recovery observed suggests that perioperative optimization strategies may play a supportive role in mitigating ischaemia‐related injury. Future prospective studies incorporating volumetric parenchymal measurements, split renal function scintigraphy and standardized functional follow‐up intervals would substantially strengthen the conclusions drawn here.

## 5 CONCLUSIONS

After minimally invasive PN, renal function demonstrates a pattern of early decline followed by substantial recovery at the 6‐month period. With proper perioperative renal‐protective strategies and limiting the WIT to a mean of under 20 min, the effect of WIT can be mitigated. Tumour size and preoperative eGFR are the main predictors of late eGFR changes.

## AUTHOR CONTRIBUTIONS

**Mohamed Elsaqa:** Conceptualization; data collection; literature review; writing original draft. **Ahmed Rabie:** Data collection; writing. **Israel Franco:** Methodology; statistical analysis; critical revision. **Andrew Fishman:** Methodology; critical revision; supervision. **Wayne Debeatham:** Data collection; supervision. **Michael Grasso:** Conceptualization; critical revision; supervision.

## CONFLICT OF INTEREST STATEMENT

The authors declare no conflicts of interest.

## INFORMED CONSENT

IRB approval was obtained (Northwell Health, No. 26‐0319). The IRB waived informed consent.

## Data Availability

The data that support the findings of this study are available from the corresponding author upon reasonable request.
